# Biosynthesis of silver nanoparticles using extract of *Rumex nepalensis* for bactericidal effect against food-borne pathogens and antioxidant activity

**DOI:** 10.3389/fmolb.2022.991669

**Published:** 2022-09-20

**Authors:** Addisie Geremew, Laura Carson, Selamawit Woldesenbet

**Affiliations:** Cooperative Agricultural Research Center, Prairie View A&M University, Prairie View, TX, United States

**Keywords:** *Rumex nepalensis*, Rn-AgNPs, antimicrobial, antioxidant, foodborne, drug-resistant

## Abstract

The evolution and incidence of multidrug-resistant food-borne pathogens still become a critical public health global issue. To avert this challenge there is great interest in medical applications of silver nanoparticles. Thus, this study aimed to synthesize silver nanoparticles (Rn-AgNPs) using aqueous leaf extract of Nepal Dock (*Rumex nepalensis* Spreng) and evaluate their antibacterial potential against food-borne pathogens and antioxidant activity. The Rn-AgNPs were characterized by UV-Vis spectrophotometry, Dynamic Light Scattering (DLS), Scanning Electron Microscopy (SEM), and Fourier Transform Infra-Red Spectroscopy (FTIR). The antibacterial activities of the Rn-AgNPs were evaluated using agar well diffusion (zone of inhibition, ZOI) and microdilution (minimum inhibitory concentration, MIC and minimum bactericidal concentration, MBC) methods. The antioxidant property of the Rn-AgNPs was investigated using radical scavenging (DPPH and hydroxyl) assays. The UV-Vis spectra of Rn-AgNPs elucidated the absorption maxima at 425 nm and FTIR detected numerous functional groups of biological compounds that are responsible for capping and stabilizing Rn-AgNPs. DLS analysis displayed monodispersed Rn-AgNPs of 86.7 nm size and highly negative zeta potential (-32.5 mV). Overall results showed that *Escherichia coli* was the most sensitive organism, whereas *Staphylococcus aureus* was the least sensitive against Rn-AgNPs. In the antioxidant tests, the AgNPs radical scavenging activity reached 95.44% at 100 μg/ml. This study indicates that Rn-AgNPs exhibit a strong antimicrobial on *L. monocytogenes, S. aureus*, *S. typhimurium,* and *E. coli* and antioxidant and thus might be developed as a new type of antimicrobial agent for the treatment of multidrug-resistant foodborne pathogens and extensible applications in nanomaterial food- and nanocomposite-based antimicrobial packaging and/or as an antioxidant.

## 1 Introduction

The evolution and prevalence of multidrug-resistant (MDR) food-borne pathogens linked with the consumption of contaminated plant and animal products become a critical public health global issue (Tanwar et al., 2014; Sevindik et al., 2017). MDR bacterial infection may lead to a paramount economic loss, an increase in mortality and morbidity rates, and a prolonged hospitalization period (Patel et al., 2008; Abebe et al., 2020). According to the WHO (2015), every year about 30% of the population in developed countries is affected by foodborne diseases. Thus, it is compelling to develop an alternative treatment to overcome drug resistance in food-borne bacterial pathogens.

In this regard, the incorporation of metallic nanoparticles into nanomedicine is gaining more attention in the discovery of an alternative treatment for MDR bacteria (Choi et al., 2021) owing to several physical and biochemical properties (Lateef et al., 2018; Bruna et al., 2021). Amongst the nanoparticles, silver nanoparticles (AgNPs) are of great significance in spectrally selective coatings, intercalation material for electrical batteries, optical receptors for bio-labeling, and antimicrobial materials in the healthcare industry (Veisi et al., 2016; Gan et al., 2018; Hemmati et al., 2018; Bruna et al., 2021). AgNPs exhibit antibacterial properties through the initiation of oxidative stress, altering membrane permeability, interaction with essential enzymes and proteins, and inhibition of DNA replication (Sre et al., 2015; Ahn et al., 2018; Loo et al., 2018; Balachandar et al., 2019; Alomar et al., 2020; Yin et al., 2020; Choi et al., 2021). However, the detailed mechanism and pathway of AgNPs role as an antimicrobial agent are still rudimentary. With the rising demand for AgNPs as efficient antimicrobial agents, efforts are being made to shift over to ecological benign and cost-effective materials for reduction of Ag^+^ ions to Ag^0^ as a replacement for chemical mediated syntheses. These syntheses employ either microbes or plants. However, plant extracts intermediated synthesis of AgNPs lessens various problems coupled with the maintenance of pure microbial cultures (Loo et al., 2012; Ahmed et al., 2016). Several studies on the green synthesis of AgNPs using plant extracts have been reported (Selvam et al., 2017; Gan et al., 2018; Hemmati et al., 2018). The principle in such approaches is that plant-assisted reduction occurs during the nanoparticles synthesis as a result of the presence of phytochemicals (Veisi et al., 2016; Barabadi et al., 2017; Ovais et al., 2018; Balachandar et al., 2019).

Additionally, medicinal plants have been the foundation of the prevention, control, and treatment of numerous ailments (Hagh-Nazari et al., 2017; Mohammed et al., 2018; Zangeneh et al., 2018; Alomar et al., 2020; Mohammed et al., 2020). They are also widely used to synthesis nanoparticles using their bioactive constituents for the development of alternative medicines (Shaikh et al., 2018; Alomar et al., 2020). *Rumex nepalensis* Spreng. (Nepal Dock) is one of the medicinal plants with a broad spectrum of therapeutic potencies in traditional medicine systems (Alberto et al., 2016; Jain and Mehata, 2017; Meresa et al., 2017; Shaikh et al., 2018; Gonfa et al., 2021). Phytochemical screening reveals that the *R. nepalensis* contains numerous constituents namely, amino acids, quercetin, alkaloid, phenols, flavonoids, triterpenoids, stilbene glycosides, tannins, saponins, resveratrol, anthraquinone, vitamin C, cardiac glycoside, sterols, steroids, sitosterols, emodin, endocrocin, chrysophanol, neopodin, physcion, torachrysone, aloesin and catechin (Ghosh et al., 2001; Mei et al., 2009; Kunwar et al., 2010; Liang et al., 2010; Anusuya et al., 2012; Solanki and Dalsania, 2012; Farooq et al., 2013; Wahid et al., 2013; Gonfa et al., 2021). Extracts and metabolites from different parts of the plant species have shown to possess purgative, antioxidant, antifungal, antibacterial, antihistaminic, anticholinergic, antibradykinin, anti-prostaglandin, antipyretic, analgesic, with a diuretic, astringent, anti-rheumatic, antiseptic, anti-allergic and anti-snake bite activities (Kumar et al., 2011; Shrestha and Timilsina, 2017; Shaikh et al., 2018; Mohammed et al., 2019; Gonfa et al., 2021; Mohammed et al., 2021). Although the phytochemical profile and ethnobotanical use of *R. nepalensis* have been well studied, the potentials of the chemical constituents in the reduction and stabilization for the synthesis of AgNPs and mode of action on MDR foodborne pathogens are unknown.

The current study aimed to synthesize and characterize AgNPs from aqueous leaf extract of *R. nepalensis* as a reducing and stabilizing agent. Furthermore, the antimicrobial and antioxidant activity of biosynthesized AgNPs against food-borne pathogens including *Staphylococcus aureus*, *Escherichia coli*, *Salmonella typhi*, and *Listeria monocytogenes* were evaluated through an *in vitro* investigation.

## 2 Materials and methods

All chemicals used were of analytic grades. Silver nitrate (Sigma Aldrich, 99%), Methanol (Sigma Aldrich, St-Louis, MO, 99%), DDPH (SRL, 99%), Hydrogen peroxide (Fischer Scientific, 30%) and Ascorbic Acid (Sigma Aldrich, St-Louis, MO, 99%) were purchased.

### 2.1 Plant samples and extract preparation

Green leaves of *R. nepalensis* Spreng (Nepal Dock) were collected from the Bill and Vara Daniel Farm and Ranch located at Prairie View A&M University (PVAMU) and the sample was identified by the plant systematist at the College of Agriculture Research Center (CARC) at PVAMU. The voucher specimen (RNTXUS-1252022) was deposited in the mini herbarium of the CARC. The collected leaves were washed with tap water to eliminate debris, followed by rinsing with distilled water. Subsequently, the leaves were freeze-dried and crushed into powder using an electrical blender. Then, fine plant powder (10g) was added to 200 ml deionized water and heated at 40 °C for 30 min. The extract solution was left to cool at room temperature and centrifuged at 8,000 RPM for 20 min twice. The supernatant was further filtered using polyvinylidene fluoride (PVDF) syringe filter (0.45 μm) and was kept at 4°C pending the synthesis of the AgNPs.

### 2.2 Biosynthesis of silver nanoparticles from the leaf extract of *R. nepalensis*

The leaves extract of *R. nepalensis,* RLE (5 ml; 58 mg/ml; pH = 4.6) was mixed with 20 ml freshly prepared 0.05 M AgNO_3_ solution in a conical flask, the mixture was heated continuously at 40°C for 10 min. A change in color of the mixture from light yellow to dark brown was considered as an indicator of the formation of *R. nepalensis* silver nanoparticles (Rn-AgNPs). Also, the bioreduction of silver ions was monitored by ultraviolet-visible (UV-Vis) spectrophotometer (SpectraMax®PLUS 384, England). The synthesized Rn-AgNPs were centrifuged at 8,000 RPM for 10 min and nanoparticles were collected after decanting the supernatant. The collected nanoparticles were stored in dark glass bottles to avoid photo-activation and degradation. Prior to further characterization of the Rn-AgNPs, the effect of physicochemical conditions such as pH, temperature and incubation period were tested and optimized in a way that one parameter was changed while the rest of the parameters were kept constant using UV-Vis. The reaction mixture was incubated at different temperatures 20°C, 30 and 40 °C as well pH of 3, 6, 7, 10 and 12. The reaction mixture was observed by the UV-Vis spectrophotometer at different time intervals (0.5, 1, 1.5, 2 and 2.5 h).

### 2.3 Characterization of the silver nanoparticles

To characterize colloidal Rn-AgNPs stability and monitor complete bioreduction of Ag^+^, ultraviolet-visible (UV–Vis) spectral analysis was carried out using a SpectraMax® PLUS 384 with a wavelength range between 200 and 750 at room temperature. The mean size and zeta potential of Rn-AgNPs were determined by dynamic light scattering (DLS) procedure operating Litesizer^TM^ 500 (Anton Paar, Austria) equipped with a 10-mW He–Ne laser (633 nm) running at an angle of 90° and a temperature of 20°C. The numbers of measurements for hydrodynamic (Z-average) size and polydispersity index (PDI) of the synthesized Rn-AgNPs were evaluated and optimized by the Kalliope Software workflow. The average of the zeta potential values was calculated by three independent measurements, each one obtained as the mean of up to 100 counts.

Fourier transform infrared (FTIR) spectra of *R. nepalensis* leaf extract and the biosynthesized Rn-AgNPs were measured with FTIR spectrophotometer (JASCO/FTIR-6300, Japan) to identify the potential functional groups responsible for the bioreduction and stability of the AgNPs with a resolution of 4 cm^−1^ at a frequency range of 4,000–500 cm^−1^. To acquire further understanding of the shape and size of the Rn-AgNPs, analysis of the sample was carried out using scanning electron microscopy (SEM) and energy-dispersive spectroscopy (EDX) techniques (JOEL JSM-6010LA, Japan). The elemental composition analysis of the lyophilized Rn-AgNPs was executed using EDX on the SEM. Moreover, the X-ray diffraction (XRD) measurement for bio-reduced Rn-AgNPs was carried out on X-ray diffractometer (XRD-7000, Shimadzu, Japan) run at 40 kV and 30 mA. The spectrum was recorded by CuKα radiation with a wavelength of 1.514 Å in the 2θ range of 10° – 80^°^. The average crystalline size was calculated using Debye- Scherrer formula:

$$D=\frac{\;k\lambda }{\beta \;\cos\theta }\;$$ (1)

where D is the average crystalline size of the nanoparticles, k is geometric factor (0.9), λ is the wavelength of the X-ray radiation source and β is the angular full-width at half maximum of the XRD peak at the diffraction angle θ (Dubey et al., 2010).

### 2.4 Antibacterial activity

#### 2.4.1 Analysis of anti-microbial sensitivity

The antibacterial activity of the Rn-AgNPs was evaluated against two Gram-negative *Escherichia coli* 0157: H7 and *Salmonella typhimurium* (ATCC 14028), and two Gram-positive bacterial strains *Staphylococcus aureus* (ATCC 12228), and *Listeria monocytogenes* (ATCC 19111) using the well-diffusion method. Bacteria were cultured in Trypticase Soy broth medium (Becton, Dickinson and Company, USA) and incubated using an Isotemp incubator (Fisher Scientific) at 37°C for 24 h. Subsequently, 100 μl of the pure cultures of bacteria were sub-cultured on Trypticase Soy Agar plates (Becton, Dickinson and Company, USA). The bacteria were swabbed onto the agar plates using a sterilized spreader. The solid medium was gently punctured with a sterile glass borer to make five wells each measuring 6 mm diameter in each plate which were equidistant from the center of the dish. Then, 25, 50, 100 and 200 μgmL^−1^ of synthesized AgNPs, 100 μgmL^−1^ of RLE, 100 μgmL^−1^ of streptomycin (positive control) and distilled water (negative control) 100 μl each, were added gradually until each hole was saturated and were incubated for approximately 24 h at 37^o^C. The inhibition zones were measured after incubation and expressed as millimeter (mm) in diameter using ProtoCOL3 (Synbiosis, Cambridge, UK).

#### 2.4.2 Determination of minimum inhibitory and bactericidal concentrations

The minimum inhibitory concentration (MIC) of the Rn-AgNPs was determined by the broth micro-dilution method. The growth in terms of cell density was then adjusted to 0.5 McFarland turbidity standard (A600 = 0.1), corresponding to 1 × 10^6^ CFU/ml. Different concentrations of Rn-AgNPs were prepared using sterilized water and added to the microtiter wells to obtain the final concentrations of 5, 10, 20, 25, 30, and 35 μg/ml. Tryptic soy broth media (100 µl), 1 × 10^6^ bacterial culture (20 µl), and different concentrations (5–200 μg/ml) of Rn-AgNPs (80 µl) were added in each well of 96-well microplate (Yehia and Al-Sheikh, 2014) to obtain a final volume of 200 µl in each well. Further, the microplates were incubated at 30°C for 24 h. For each dilution series, equal volume (80 µl) of the test sample (AgNPs) and Tryptic soy broth (no bacterial inoculums) served as negative control while untreated cell suspensions (only medium and bacteria inoculums) were employed as positive controls. The MIC values were identified as the minimum concentration at which no visible bacterial growth was recorded. The MIC of an antibacterial agent for a particular bacterium is defined as its concentration in the growth medium which causes complete inhibition of bacterial growth without cell killing even after overnight incubation. Minimum bactericidal concentrations (MBC) of the tested samples were evaluated by sub-culturing about 25 µL of wells with a concentration equal to or higher than MIC on Tryptic Soy Agar plates and incubated at 30°C for 24 h. The lowest concentration that did not show bacterial growth was defined as the MBC value. All the experiments were carried out in triplicate.

### 2.5 Antioxidant activity

#### 2.5.1 Hydrogen peroxide scavenging assay

Hydrogen peroxide scavenging potential of *R. nepalensis* extract and Rn-AgNPs was assessed following a slightly modified method of Patel et al. (2010). In this test, H_2_O_2_ (40 mM) was prepared freshly in 0.1M phosphate buffer solution (PBS) (pH 7.4). Of this, 0.7 ml was added to reaction tubes containing 5 ml of PBS with required quantities of Rn-AgNPs (20, 40, 60, 80 and 100 μg/ml) dissolved in 50/50 volume mixture of methanol and water and incubated for 10 min. The mixture was vortexed, and after 10 min the optical density was recorded at 230 nm using a UV-Vis spectrophotometer (SpectraMax®PLUS 384). Ascorbic acid was used as a standard while phosphate buffer (40 mM, pH 7.4) was used as blank. The percentage of H_2_O_2_ scavenging activity was computed as:

$$\%\;Radical\;scavenging\;\left(Hydrogen\;Peroxide\;\right)=\frac{\left(Ao-A1\right)}{Ao}X100\;$$ (2)

where A_0_ and A_1_ denoted the optical density (OD) of the ascorbic acid and the Rn-AgNPs or the leaf extract (RLE), respectively. Using the regression equation of the plot between scavenging activity % and Rn-AgNPs concentration, the IC_50_ value was determined.

#### 2.5.2 DPPH radical scavenging activity

The DPPH assay was performed to test the free radical scavenging activity of the leave extract, Rn-AgNPs, and standard ascorbic acid using the stable radical 2, 2-diphenyl-1-picrylhydrazyl (DPPH) with a modification of Choi et al. (2002). In each well of a 96-well microplate, 30 μl of different concentrations (20, 40, 60, 80 and 100 μg/ml) of the leave extract and Rn-AgNPs, separately, were mixed with DPPH radical solution in methanol (0.1 mM, 170 μl). The plate was draped with aluminum foil and incubated at room temperature in the dark for 30 min. Then the absorbance was recorded at 517 nm using UV-Vis spectrophotometer (SpectraMax® PLUS 384) according to (Choi et al., 2002). DPPH methanol reagent without the leave extract and Rn-AgNPs was used as control and percentage of inhibition was calculated by the following formula:

$$\%\;Radical\;scavenging\;\left(DPPH\right)=\frac{\left(Ao-A1\right)}{Ao}X100\;$$ (3)

where A_0_ and A_1_ represent the OD of the/ascorbic acid, the RLE or Rn-AgNPs respectively. A graph was plotted with % scavenging activity versus Rn-AgNPs concentrations, and the equation obtained was used for the calculation of IC_50_.

### 2.6 Effect of Rn-AgNPs on leakage of bacterial cell membrane

The leakage of proteins through the membrane was determined using *E. coli* 0157:H7*, S. typhimurium* ATCC 14028, *S. aureus* ATCC 12228, and *L. monocytogenes* ATCC 19111 cultured in Trypticase Soy broth medium. mL sample was drawn from each culture and was marked as 0 h sample. One milliliter of Rn-AgNPs solution (1 mg/ml) was added to each culture and was incubated at 200 rpm and 37°C. The sample was withdrawn after 2, 4, and 6 h from each culture. All the samples were centrifuged at 8,000 rpm for 5 min. Pellet was removed and the supernatant was stored at −30°C immediately, and then the concentration of proteins was determined by Bradford’s method immediately.

### 2.7 Statistical analysis

Data related to the MIC, MBC, membrane protein leakage and antioxidant activities were subjected to analysis of variance (one-way ANOVA) SPSS (2008; version 17.5) statistical software. Tukey multiple comparison test was used to calculate the differences between each factor (RLE, Ascorbic acid and Rn-AgNPs) for antioxidant activities. The differences with *p* < 0.05 were considered significant. All generated data are from at least three replicates and expressed as mean ± SE.

## 3 Results and discussion

Advances in nanomedicine have led to great prospects for the synthesis of nanoparticles using biogenic agents with unique characteristics that can combat multidrug-resistant (MDR) bacteria. In the present study, we developed silver nanoparticles and investigated their antibacterial and antioxidant activity against foodborne bacteria as part of efforts to develop alternative agents for MDR bacteria.

### 3.1 Biosynthesis and characterization of silver nanoparticles (AgNPs)

**FIGURE 1:**
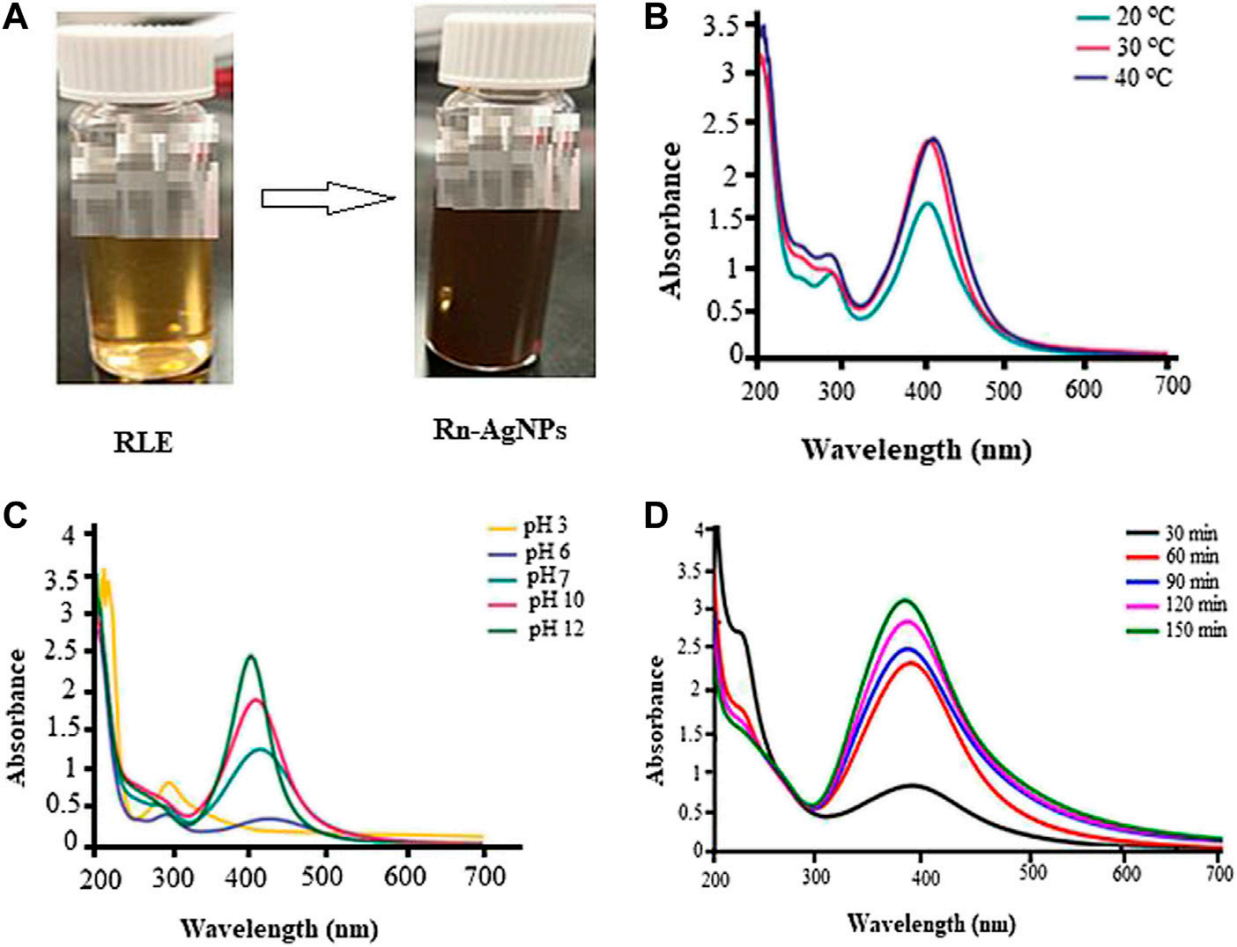
Biosynthesis and UV-Vis absorbance spectra of Rn-AgNPs under different reaction conditions) **(A)** coloration images, **(B)** pH, **(C)** temperature and **(D)** reaction time in colloidal state.

When the fresh *R. nepalensis* leaf extract (RLE) was mixed with AgNO_3_ solution, the reduction of silver ion was reflected in a color change from pale yellow to colloidal dark brown (Figure 1A). The progressive color change indicates the formation of Rn-AgNPs. The color change could be related to the excitation of surface plasmon resonant (SPR) oscillation of free metal electrons under the irradiation of an electromagnetic wave of the produced nanoparticles (Honary et al., 2013; Banerjee et al., 2014; Balachandar et al., 2019; Algebaly et al., 2020; Alomar et al., 2020). The secondary metabolites in plant extracts can act as natural capping and reducing agents during the green synthesis of AgNPs. The leaf extract of *R. nepalensis* proved to be an enriching source of phytochemicals (Gonfa et al., 2021) that potentially reduced Ag^+^ to Ag^0^ followed by agglomeration and stabilization of Rn-AgNPs**.**

UV-vis spectrum of samples in the region of 200–700 nm was used to further detect the synthesis of Rn-AgNPs. The UV–vis spectra of synthesized Rn-AgNPs exhibited an absorption peak at about 425 nm (Figure b-c), which is mainly due to SPR of the nanoparticles (Aygun et al., 2020; Wei et al., 2020; Hu et al., 2022). Corresponding with Mie theory (Javed and Mashwani, 2020), spherical nanoparticles display only a single SPR band. Then, it can be concluded that biosynthesized Rn-AgNPs are unanimously spherical in nature. However, arrays of physicochemical variables including pH, temperature, reaction time and reactants concentration profoundly influence the size, shape and morphology of AgNPs (Wei et al., 2020; Wei et al., 2021; Hu et al., 2022). In the present study, adjusting the pH from the acidic to the neutral and then alkaline conditions increased the synthesis of the Rn-AgNPs and detection of the highest peak at pH 12 (Figure 1B). pH plays a substantial role in the fabrication of AgNPs by increasing or decreasing the concentration of H^+^ ions that modulate the electronegative state of the phytometabolites which serve as capping agents and are responsible for the reduction of AgNO_3_ (Khan et al., 2019; Mathew and Thomas, 2019; Ahmed and Mustafa, 2020; Javed and Mashwani, 2020). In agreement with our results, Khalil et al. (Khalil et al., 2014) showed that the rate of formation of AgNPs increased at high pH. The SPR band was also shifted toward a lower wavelength from 435 to 425 nm which displays the synthesis of the small size of Rn-AgNPs. Such symmetrical and narrow SPR band under high pH signifies the fabrication of small, spherical and monodisperse nanoparticles. In contrast, a very broad peak noted at acidic pH represents anisotropic, large size and aggregated polydisperse nanoparticles (Khan et al., 2019; Javed and Mashwani, 2020). Temperature is one of the factors influencing the synthesis, shape, size and biological activities of AgNPs (Javed and Mashwani, 2020; Wei et al., 2021; Hu et al., 2022). Intriguingly, Rn-AgNPs were synthesized at 20, 30 and 40°C, and a characteristic SPR band was observed for Rn-AgNPs synthesized at 40°C (Figure 1C). In this particular study, we were investigating syntheses at low temperatures for optimal green synthesis of Rn-AgNPs but not the maximum threshold temperature. Hence, very high temperatures can alter plant metabolites and functional conformations and then ultimately hinder the synthesis of AgNPs (Wei et al., 2015; Javed and Mashwani, 2020). The lack of significant shift of the absorption peak either towards a longer or shorter wavelength at 30 and 40°C suggests that the size of synthesized nanoparticles was stable. UV–vis spectrophotometer showed the highest OD after 1.5 h of the incubation of the reaction mixture (Figure 1D). Thus, the reaction time is a vital factor in modulating the size and shape of Rn-AgNPs (Mata et al., 2015; Ahmed et al., 2016; Al-Salhi et al., 2019; Javed and Mashwani, 2020).

**FIGURE 2:**
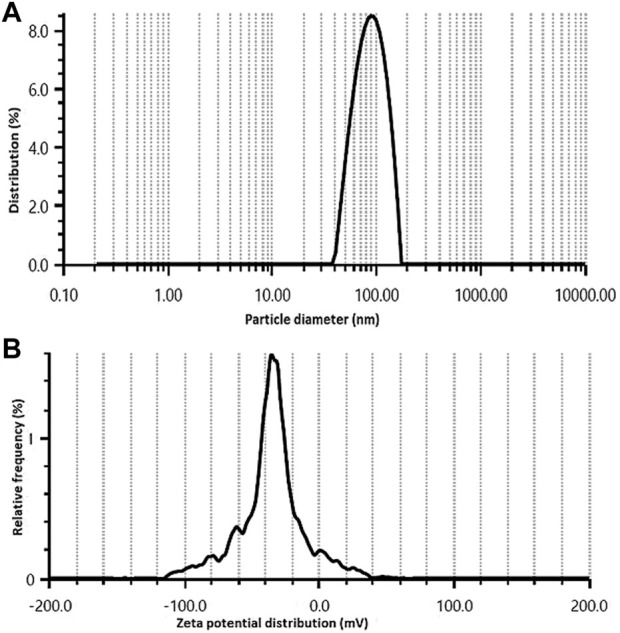
Size distribution **(A)** and zeta potential **(B)** of Rn-AgNPs obtained using aqueous extracts of *R. nepalensis*.

The size distribution of synthesized Rn-AgNPs was analyzed using the DLS technique and revealed a mean size of 86.7 ± 2.6 nm with a monodispersed peak (Figure 2A) and a polydispersity index (PDI) of 0.24. It collectively represents the size of the metallic core and the biological shield that protects the AgNPs from agglomeration and provides surface modifications. The polydispersity of the Rn-AgNPs (0.24) recorded exhibits that these nanoparticles are well separated and have narrow dispersity to be used as biologically effective agents. The small size (86.7 ± 2.6 nm) and dispersity of the Rn-AgNPs the particles are well surfaced by secondary metabolites which prevent agglomeration and provide them with the characteristics such as large surface area and potential biochemical attributes (Ahmed et al., 2016; Duran et al., 2016; Deshmukh et al., 2019). Also, the zeta potential of the fabricated nanoparticles dispersed in water at ambient temperature showed negative values of -32.5 ± 1.5 mV **(** Figure 2B), indicating a negative surface charge. The negative zeta potential value of Rn-AgNPs could be attributed to negatively charged capping agents attached to the surface of nanoparticles (Wei et al., 2020).

**FIGURE 3:**
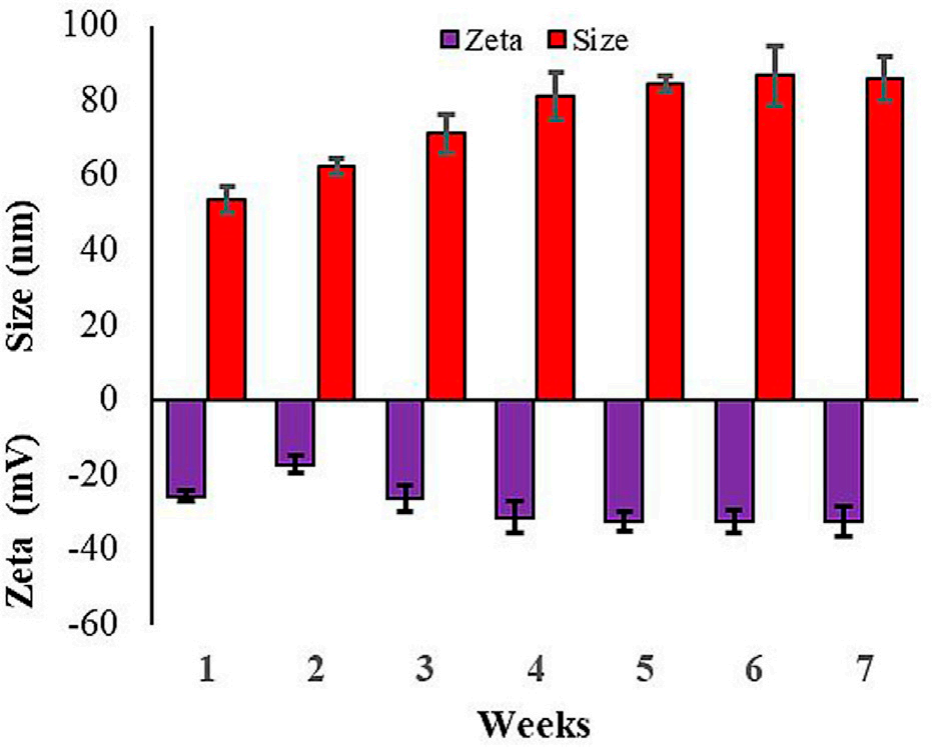
Variation in particle size distribution and zeta potential of Rn-AgNPs across time.

Further stability of Rn-AgNPs size and surface charge were characterized using a zeta sizer across time. The hydrodynamic diameter and surface charge of Rn-AgNPs in a difference of 1 week showed fluctuation in size and zeta potential (size = 86.73 nm, ζ = −32.5 mV) become stable after 4 weeks in the colloid state (Figure 3). Under the ambient condition, the average size of as-synthesized Rn-AgNPs increased from 53.41± 1.5 nm to 86.73 ±7.99 nm. The value of zeta potential is zero at the iso-electric point confirming its instability, whereas the highly negative and positive zeta potential (Kuznetsova and Rempela, 2015), validates the high stability (−32.5 mV) of synthesized Rn-AgNPs in colloidal suspension.

**FIGURE 4:**
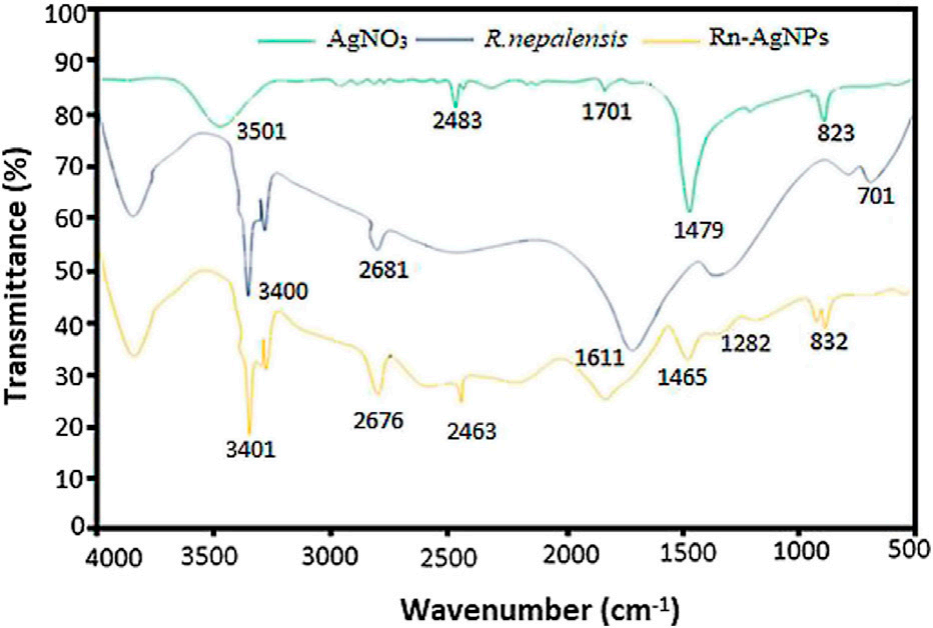
Comparison of the FTIR spectra of the *R. nepalensis* extract, AgNO_3_ and Rn-AgNPs. Each peak in the Rn-AgNPs indicates the functional group of the phytochemical involved in the nanoparticle synthesis.

FTIR measurements were carried out to identify the major functional groups in the leaf extract and their possible involvement in the synthesis and stabilization of silver nanoparticles. The FTIR spectra of the *R. nepalensis* leaves extract (RLE) and the synthesized Rn-AgNPs are shown in Figure 4. The peculiar peaks around 3,401 cm^−1^ were associated with amine (N-H) and alcohol (O-H) stretching bands (Barabadi et al., 2018). AgNPs exhibited C–H stretching vibration at 2,676 cm^−1^ and C=C-C stretching vibration of aromatic rings at 1,611 cm^−1^ (Barabadi et al., 2017). Also, the characteristic peaks around 1,465 cm^−1^ and 1,282 cm^−1^ were assigned to heterocyclic vibration (polyphenols) and carbonyls (C=O), respectively (Hu et al., 2022). Additionally, the characteristic peaks at 832 cm^−1^ were attributed to alkyne C-H bending vibration (Balachandar et al., 2019). The FTIR spectra of the leaf extract and the synthesized AgNPs presented highly similar peaks with a small shift in both spectra, indicating that the synthesized Rn-AgNPs possess bioactive molecules from the RLE. These functional groups may act as reducing and capping agents on Rn-AgNPs and were responsible for the reduction of silver ions (Ahmed et al., 2016; Balachandar et al., 2019). Several phytochemicals such as phenols, flavonoids (e.g., catechin, quercetin, etc.), anthraquinones, naphthalenes, saponins, glycosides, triterpenoids, tannic acid, sterols, and sitosterols reported in *R. nepalensis* (Gonfa et al., 2021) can effectively act as reducing and stabilizing agents (Valsalam et al., 2019; Alarjani et al., 2022). For example, studies have shown that quercetin reacts with Ag^+^ through OH groups, then reduces Ag^+^ to AgNPs and provides stability (Borodina and Mirgorod, 2014; Jain and Mehata, 2017; Badmus et al., 2020).

**FIGURE 5:**
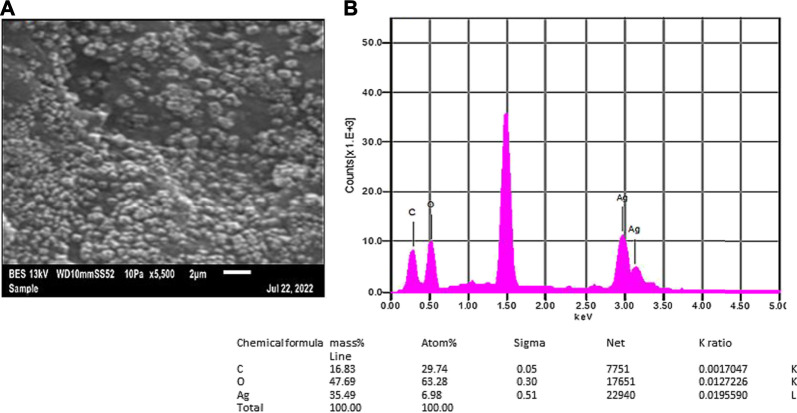
SEM micrographs **(A)** and EDX spectra **(B)** of synthesized Rn-AgNPs using *R. nepalensis* leaf extract as a reducing agent. Tabulated values represent the proportion of Ag, C and O.

The lyophilized Rn-AgNPs were analyzed for shape, size, and elemental composition under the SEM equipped with energy-dispersive X-ray spectroscopy (EDX). SEM images have demonstrated that all synthesized Rn-AgNPs were predominantly spherical in shape with an average size of about 62.85 ± 1.35 nm (Figure 5A). However, during evaporation of solvent from nanoparticles, some particles agglomerated and appeared in larger-sized nanoparticles. The EDX analysis of the synthesized Rn-AgNPs showed strong signals of counts at 3 keV of silver, whereas signals were also recorded for other elements including oxygen and carbon (Figure 5B). This absorption peak confirmed the existence of AgNPs (Rasheed et al., 2017). EDX elemental analysis substantiates the occurrence of AgNPs in pellets obtained and the presence of other elements attaching to AgNPs indicated the plant-based origin of these biomolecules. The carbon and oxygen elements detected may come from phytochemical components, which were bonded to the surfaces of the AgNPs.

**FIGURE 6:**
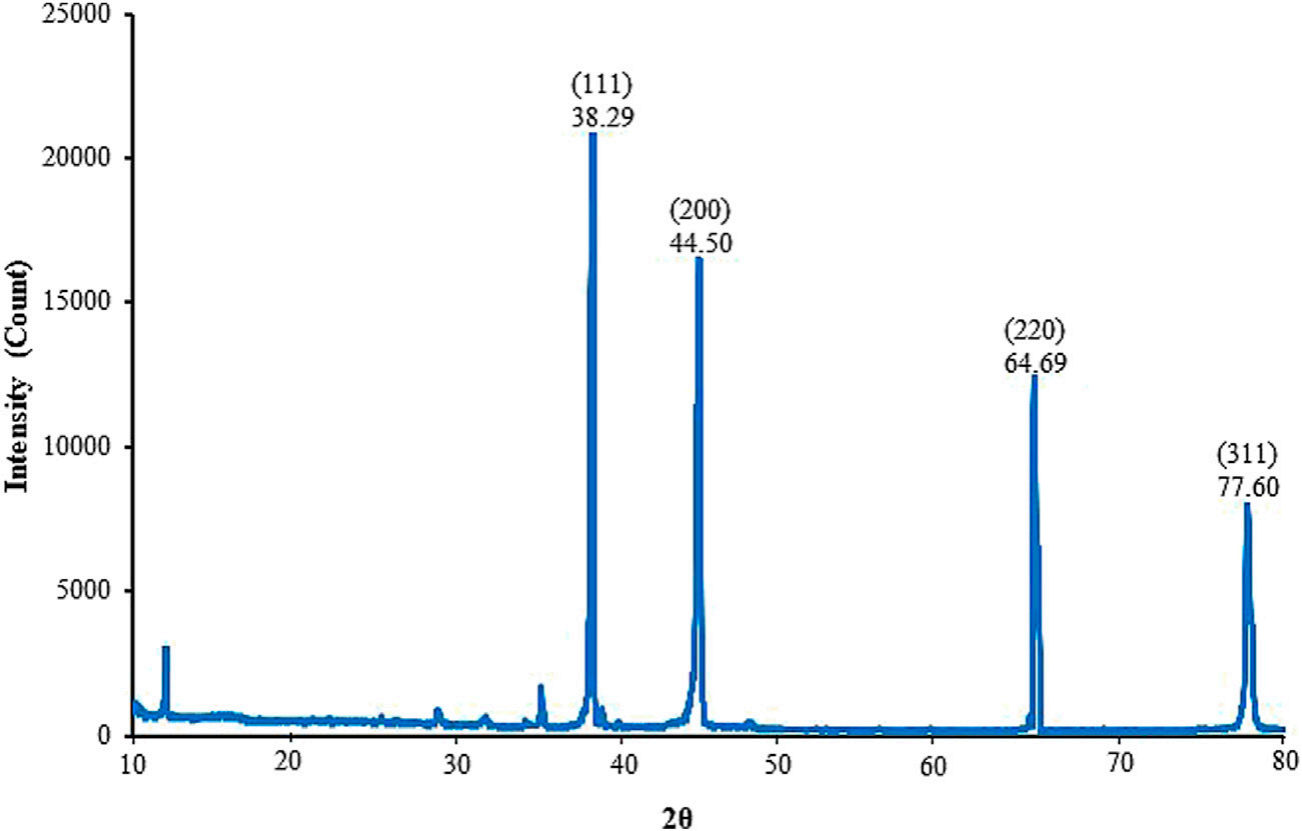
X-ray diffractometer patterns for biosynthesized Rn-AgNPs using *R. nepalensis* leaf extract.

The XRD pattern of the synthesized AgNPs is shown in Figure 6. X-ray diffraction analysis displayed four distinct Bragg reflection values of AgNPs; 38.29°, 44.50^°^, 64.69^°^ and 77.60^°^, which correspond to 111, 200, 220 and 311, indicating reflecting face-centered cubic crystalline structure of the Rn-AgNPs. The strong peak at 38.29^°^ signifies a high level of crystallinity (Pattanayak et al., 2017). The high peak at 44.50^o^ in the XRD spectra could be due to the presence of phytochemical compounds in the RLE crusting the surface of the synthesized Rn-AgNPs and stabilizing it (Krishnaraj et al., 2010). Other minor peaks detected in the XRD spectra might be associated with some organic compounds deriving from RLE (Kamaraj et al., 2017; Kumar et al., 2018). The average crystallite size of Rn-AgNPs was ∼61 nm.

### 3.2 Antimicrobial activity of the AgNPs

**FIGURE 7:**
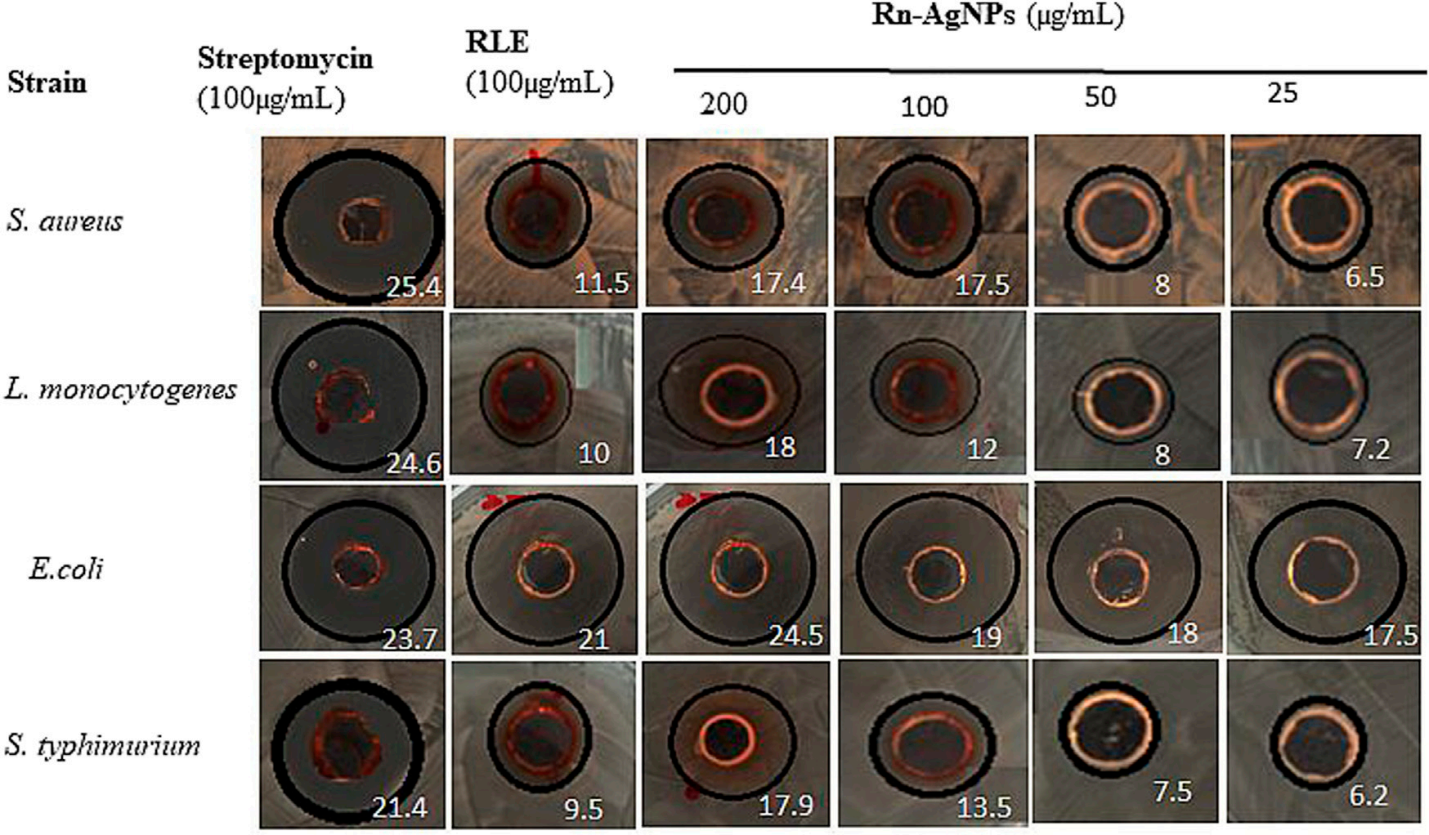
Zone of inhibition (mm) observed when gram-positive and gram-negative bacterial strains are treated with RLE and Rn-AgNPs in different concentrations. Values in white color represent zone of inhibition.

Foodborne diseases caused by drug-resistant pathogenic bacteria are continuously a critical threat to public health through worldwide communities (Tanwar et al., 2014; Sevindik et al., 2017). To avert this challenge the antimicrobial activity of Rn-AgNPs was investigated against *L. monocytogenes, S. aureus*, *S. typhimurium,* and *E. coli* using the agar well diffusion assay. The zones of inhibition, (ZOI) around each well containing Rn-AgNPs and RLE are represented in Figure 7. Among the tested bacteria, *S. aureus* was found to be most resistant with a minimum inhibition zone of 17.4 mm for Rn-AgNPs at 200 μg/ml. On the other hand, *E*. *coli* was the most susceptible bacteria with 24.5 and 17.5 mm ZOI at 200 and 25 μg/ml, respectively. Based on the overall results obtained from the ZOI data, the pattern of sensitivity was observed in the order as *E. coli* > *S. typhimurium* > *L. monocytogenes* > *S. aureus*. Enhanced antibacterial activity of Rn-AgNPs compared to RLE is coupled with their large surface area that provides more surface contact with bacteria (Logeswari et al., 2015) and significant dissolution power to release Ag^+^ ions for microbial disruption (Mohammadlou et al., 2016). An additional critical reason for the augmented antibacterial activity of AgNPs is the synergistic effect between particles and phytochemicals (Duran et al., 2016).

**FIGURE 8:**
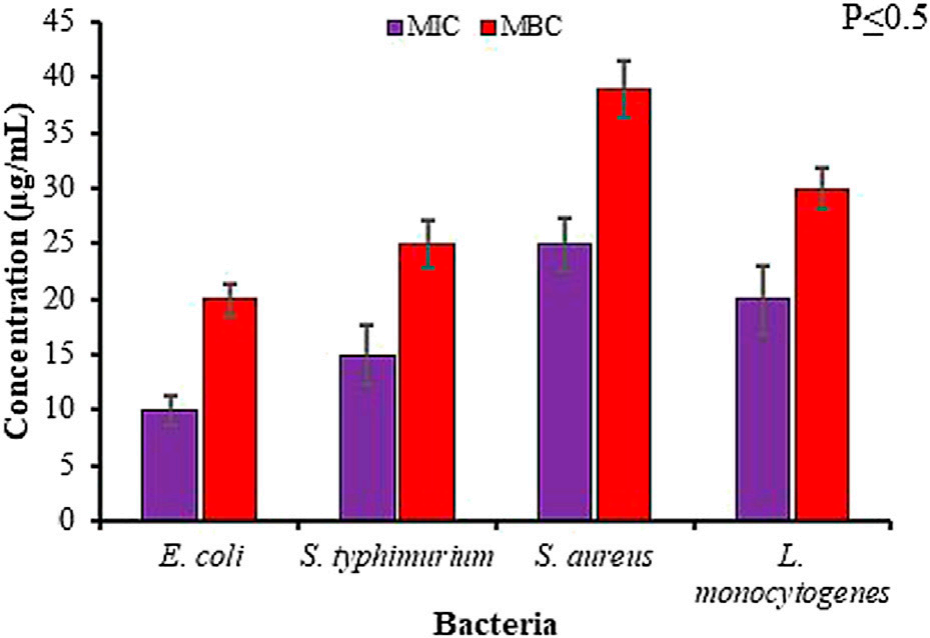
Antibacterial activity of green synthesized Rn-AgNPs measured in terms of minimum inhibitory/bactericidal concentration (MIC/MBC) for different bacterial strains.

**FIGURE 9:**
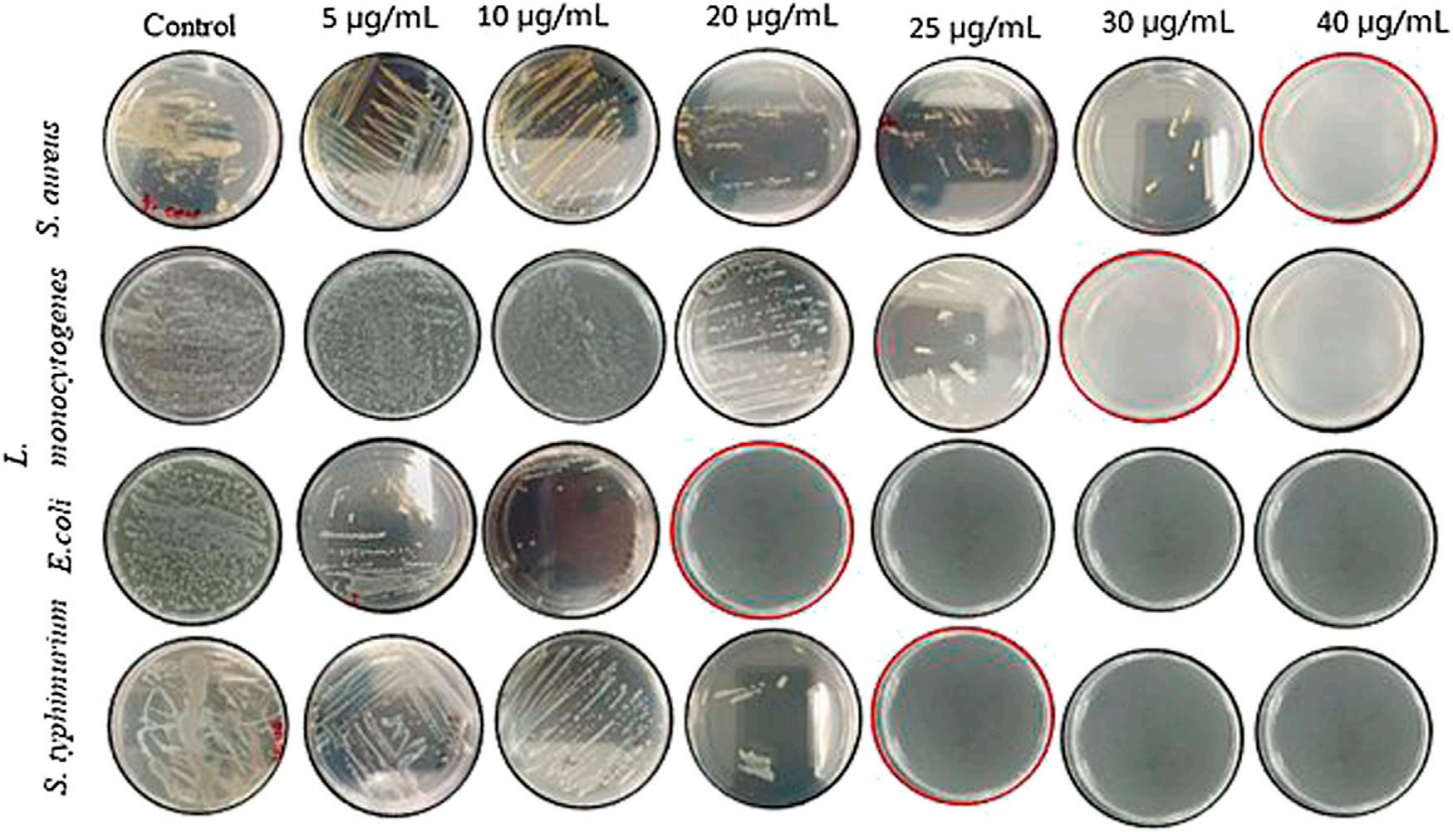
Minimum bactericidal concentration (MBC) of Rn- AgNPs against different bacterial strains. Plates with red margins show the MBC value for each bacterial strain.

Further validation of antimicrobial activity of synthesized Rn-AgNPs through well diffusion assay involved studies of MIC and MBC of Rn-AgNPs against the foodborne pathogens using the broth dilution method. The MIC was established as the lowest concentration at which no visible growth of the foodborne pathogen was observed. For gram-negative bacteria, the MIC varied from 10 to 15 μg/ml, whereas for gram-positive bacteria, it was found between 20 and 25 μg/ml (Figures 8, 9). In agreement with the ZOI, overall, the MIC and MBC values showed that gram-positive bacteria are less susceptible than gram-negative bacteria (Rautela et al., 2019). The highest value of MIC (25 μg/ml) was observed with *S. aureus*, whereas the lowest MIC (10 μg/ml) was observed with *E. coli*. Similarly, for MBC, the highest value (40 μg/ml) was observed with *S. aureus*, whereas the lowest value (20 μg/ml) was observed with *E. coli* (Figure 9)*.* Overall, Rn-AgNPs synthesized from RLE revealed broad-spectrum antibacterial properties thus, suggesting a valuable alternative treatment for foodborne pathogens. However, it was noted that gram-positive bacterium showed less susceptibility to Rn-AgNPs than most gram-negative bacteria may be due to the complex thick peptidoglycan layer of gram-positive bacteria that makes it difficult for the penetration of AgNPs into the bacterial cytoplasm (Krishnaraj et al., 2010; Vellora et al., 2013; Baygar et al., 2019). This may also be attributed to the presence of a negatively charged peptidoglycan layer that prevents the free entry of silver ions into the bacteria (Baygar et al., 2019). On the other hand, the high ZOI and low MIC and MBC by gram-negative bacteria such as *E. coli* and *S. typhimurium* indicate their sensitivity to Rn-AgNPs because of the negatively charged lipopolysaccharides that promote nanoparticles adhesion (Pal et al., 2007; Netala et al., 2014) and the presence of porins that facilitate Rn-AgNPs entrance (Chauhan et al., 2016; Rashid et al., 2017).

Despite variation in phytochemical composition in line with our results, green synthesized nanoparticles using conspecific plant species (e.g., *Rumex dantatus, Rumex hastatus,* and *Rumex scutatus*) have been shown bactericidal action against various drug-resistant bacterial pathogens (Ahmed et al., 2016; Rashid et al., 2019; Mani et al., 2021; Unal et al., 2022). However, the antibacterial efficacy of AgNPs is a function of phytochemicals acting as reducing and stabilizing agents (Ovais et al., 2018), pH, temperature, test microorganisms, concentration and their size and shape (Raza et al., 2016; Tang and Zheng, 2018; Hu et al., 2022). Moreover, studies have also shown several compounds with antifungal, anti-inflammatory, antioxidant and antibacterial activities including chrysophanol, resveratrol, orientaloside, nepodin, orcinol-glucoside, physcion, aloesin rumexoside, torachrysone, emodin, chrysophanol-8-O-β-D-glucopyranoside, emodin-8-O-β-D-glucopyranoside and nepodin-8-β-D-glucopyranoside isolated from *R. nepalensis* [(Ghosh et al., 2003; Tonny et al., 2017). Of these phytochemicals emodin obtained from other Rumex species has been also reported to have antibacterial potential against *S. aureus* and *E. coli* (Ghosh et al., 2003; Li et al., 2016) which could also be the case in the present study.

**FIGURE 10:**
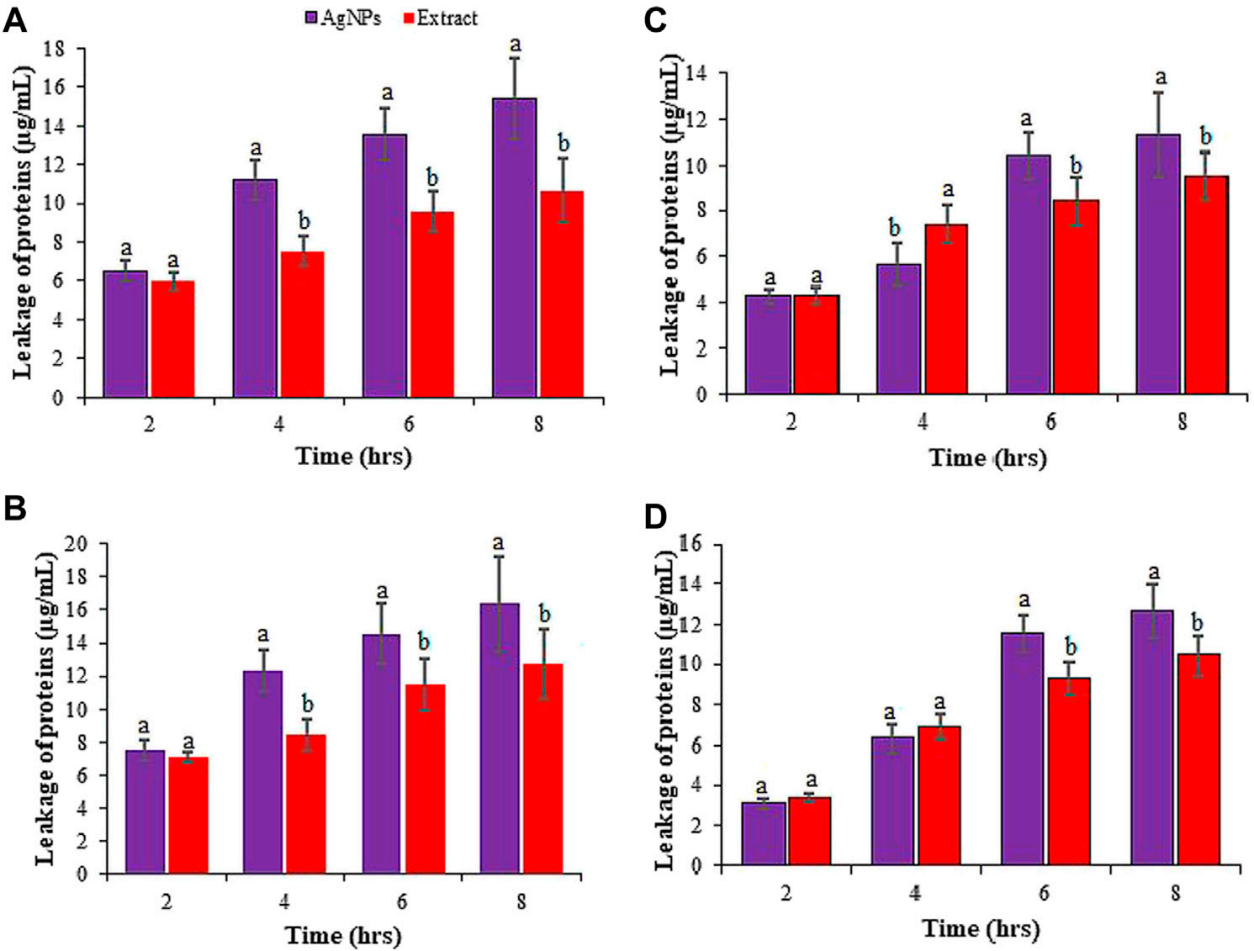
Leakage of proteins from **(A)** *E.coli*, **(B)** *S. typhimurium*, **(C)** *S. aureus*, and **(D)** *L. monocytogenes* cells treated with Rn-AgNPs. Different letters denote significant difference (*p* ≤ 0.05) between AgNPs and RLE at that particular time.

The antimicrobial effect of AgNPs has been broadly studied, yet their actual mechanisms of action is not fully understood. However, researchers proposed different conceivable mechanisms involving disruption of DNA replication and ATP production, stimulation of oxidative stress via the production of free radicals and modulation of signal transduction pathways, and damage to bacterial cell membranes leading to cell lysis (Dakal et al., 2016; Huang et al., 2020; Alvarez-Chimal et al., 2021). As the synthesized Rn-AgNPs are small and spherical, these might easily cross the bacteria cell wall resulting in destruction of the cells. Nanoparticles with spherical shape and small size provide opportunities for interactions with the bacterial cell consequently leading to increased membrane permeability and cell destruction (Pal et al., 2007; Singh et al., 2016; Kumar et al., 2021). Zhang et al. (2016) have also indicated that spherical AgNPs are more effective antimicrobial than rod-shaped ones. AgNPs disturb the membrane integrity and leakage of pathogenic bacteria as reported earlier by many researchers (Pal et al., 2007; Srikar et al., 2016). To gain more insight into the effects of Rn-AgNPs, we quantified the amounts of protein that were released by microbes. Despite the difference in bacterial types, our results indicated that Rn-AgNPs indeed caused significant loss of intracellular proteins from resting cells across different time points (Figure 10). The maximum amount of protein was released by the gram-negative bacteria *E. coli* (15.37 ± 2.1 μg/ml) and *S.* t*yphimurium* (16.37 ± 2.84 μg/ml) when treated with Rn-AgNPs. Protein leakage recorded for all bacteria was statistically significant (*p* ≤ 0*.*05) after 4, 6, and 8 h incubation. The amounts of protein were higher as compared with the RLE in all the cases, which suggests that AgNPs may have expedited the leakage of proteins from the cytoplasm of microorganisms (Figure 10). The leakage of intracellular proteins upon exposure of susceptible bacteria to Rn-AgNPs often has been invoked as evidence of membrane damaging action. AgNPs inactivate membrane-bound enzymes and proteins by interacting with disulfide bonds and blocking active sites, consequently influencing membrane function, permeability, and loss of membrane integrity (Holt and Bard, 2005; Emmanuel et al., 2017; Ovais et al., 2017; Rautela et al., 2019). In addition, AgNPs are known to inhibit the absorption of phosphorus, alter the levels of phosphate, and regulate phosphorylation in the bacterial cell (Hamouda and Baker, 2000). Furthermore, bactericidal mechanism of biogenic AgNPs is ascribed to a disruption in the cell membrane along with the formation of reactive oxygen species (ROS) that trigger enzymes denaturation, DNA damage, and ultimate cell death (Reidy et al., 2013; Duran et al., 2016; Singh et al., 2016; Saravanan et al., 2018; Aguirre et al., 2020; Kup et al., 2020). The presence of elemental oxygen in our EDX analysis may contribute to generating the ROS that disrupt the membrane ionic balance and eventually results in cell death.

### 3.3 Antioxidant activity of Rn-AgNPs

**FIGURE 11:**
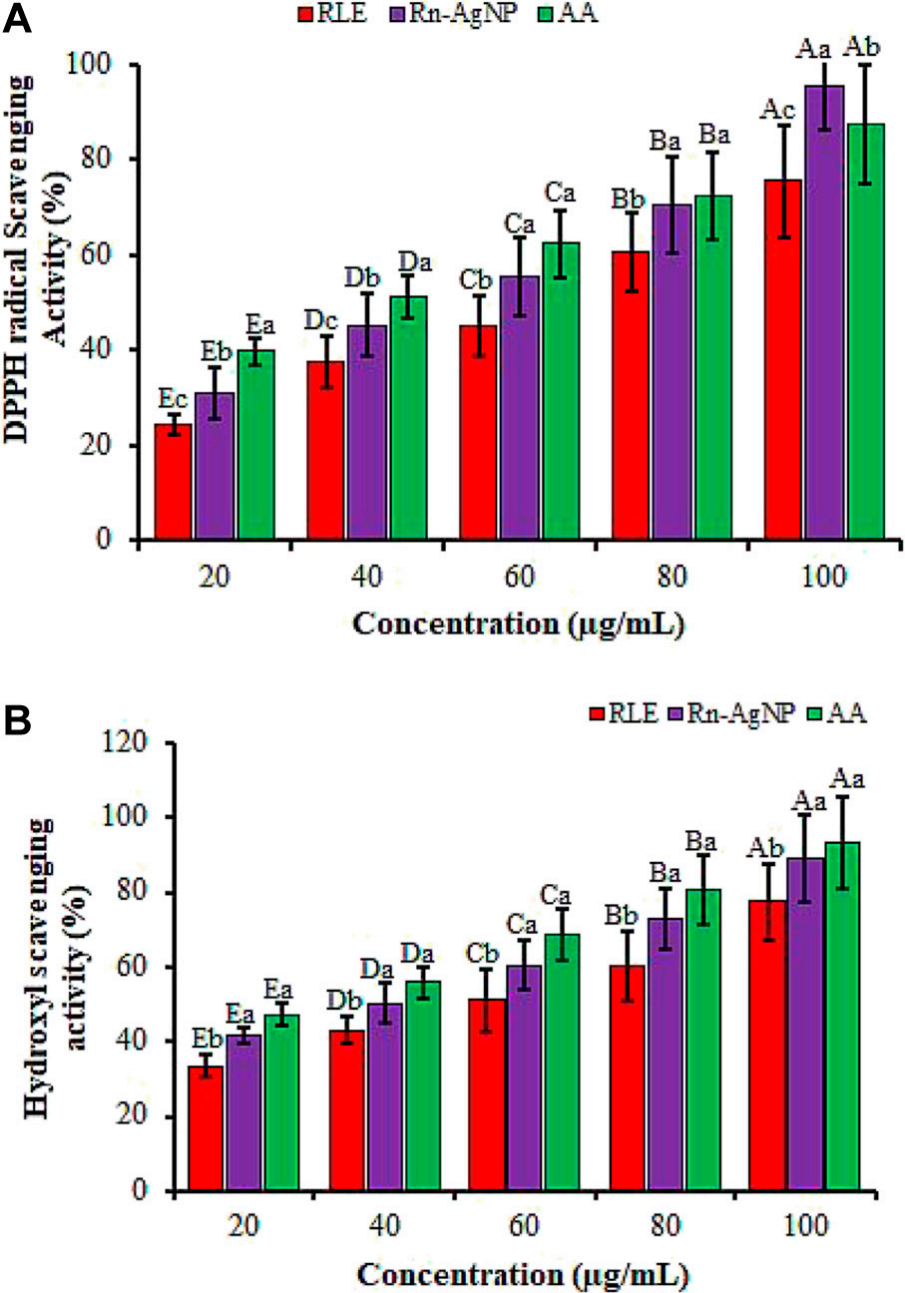
The antioxidant activity of RLE and Rn-AgNPs measured using DPPH radical scavenging (a) and hydroxyl scavenging assays. AA, Ascorbic acid. Different lower-case letters denote significant differences (*p* ≤ 0.05) among ascorbic acid, AgNPs and RLE at a particular concentration. Whereas uppercase letters denote significant differences across different concentrations for a particular antioxidant.

Oxidative stress due to cellular oxidation resulting in the generation of free radicals and reactive oxygen species (ROS) has been implicated in several ailments encompassing rheumatoid arthritis, diabetes, cancer, aging, atherosclerosis, cardiovascular diseases, and neurodegenerative diseases (Sevindik, 2019; Sevindik, 2020; Vieira et al., 2020; Hailan, et al., 2022). These radicals in excess quantities cause a damaging effect on defensive antioxidant enzymes eventually leading to cellular injury or apoptosis (Shankar et al., 2005). Here, we instigated the free-radical quenching potential of AgNPs developed from the RLE using DPPH and hydrogen peroxide scavenging assays. The DPPH radical scavenging activity of Rn-AgNPs was described in Figure 11A. The DPPH radical scavenging activities reached 30.8%, 55.8% and 70.47% at 20, 60 and 80 μg/ml respectively. However, the DPPH radical scavenging activity reached 95.44% at 100 μg/ml AgNPs and relatively exceeded the activity of the standard (87.32%). The results showed that Rn-AgNPs possessed higher antioxidant activities than RLE. The concentrations of the Rn-AgNPs and RLE able to scavenge 50% of the DPPH (EC_50_) were 39.14 μg/ml and 59.37 μg/ml, respectively. The hydroxyl scavenging activities of Rn-AgNPs and RLE ranged from 41.56% to 89.21% and from 33.54% to 77.41%, respectively with concentrations of 20–100 μg/ml (Figure 11B). As demonstrated in FTIR, the high antioxidant properties of Rn-AgNPs might be owing to the occurrence of various types of active molecules adhered on the surfaces of AgNPs as capping agents. In agreement with our results, RLE has been reported to be rich in phenols, flavonoids, anthraquinones, naphthalenes, saponins, stilbene, triterpenoids, anthraquinone glycosides, tannic acid and sterols, saponin and sitosterols (Mei et al., 2009; Raju et al., 2010; Vasas et al., 2015; Gonfa et al., 2021) of promising antioxidant agents. In addition, these active compounds not only play critical roles in the bio-reduction of Ag ions but also can scavenge free radical species through the donation of electrons or hydrogen atoms from the biomolecules attached to AgNPs (Bindu et al., 2019; Kup et al., 2020; Yousaf et al., 2020; Alarjani et al., 2022). The stronger radical scavenging activity of Rn-AgNPs might be credited to the phenolic content and the presence of nepodin in *R. nepalensis* leaf extract (Raju et al., 2010), which revealed better radical scavenging activities than ascorbic acid (Gautam et al., 2010).

## 4 Conclusion

In conclusion, the synthesized silver nanoparticles from the Himalayan dock (*R. nepalensis*) through an eco-friendly approach showed significant antibacterial activity against foodborne pathogens and increased antioxidant activity than its RLE. Thus, Rn-AgNPs might be an ideal option to develop as an antibacterial agent against drug-resistant strains of bacteria. Despite the strong antibacterial and antioxidant activity of AgNPs, further investigations are required to examine the bactericidal mechanisms at a molecular level for extensible applications in nanomaterial food- and nanocomposite-based antimicrobial packaging that can protect against food spoilage and enhance the shelf-life of the food.

## Data Availability

The raw data supporting the conclusion of this article will be made available by the authors, without undue reservation.
